# Supplementary material for: GmNMHC5, A Neoteric Positive Transcription Factor of Flowering and Maturity in Soybean
Source: Plants (Basel). 2020 Jun 25;9(6):792. doi: 10.3390/plants9060792 (PMC7356762; doi:10.3390/plants9060792)
Supplement: Supplementary file 1 [file plants-09-00792-s001.zip › Supplemental files/Figure S1 Frameshift mutations at the target sites of GmNMHC5.docx]

**Text S1 Frameshift mutations at three target sites of *GmNMHC5* generated premature translation termination codons (PTCs)**

**CDS of *GmNMHC5* (WT, wild-type)**

ATGGGGAGAGGTAAGATTGCGATTCGAAGGATCGACAACTCCACTAGCCGGCAAGTGACTTTCTCAAAGAGAAGAAATGGATTGCTGAAGAAAGCTAGAGAATTATCAATTCTTTGTGATGCTGAAGTTGGATTGATGGTGTTCTCCAGCACTGGGAAGCTTTATGACTATGCAAGCACAAGCATGAAAGCGGTTATTGAACGCTACAACAAGCTAAAAGAGGAAACCCATCACCTCATGAATCCGGCTTCAGAAGAGAAGTTTTGGCAGACAGAAGCAGCAAGCTTGAGGCAGCAGCTTCAGTACTTGCAAGAATGCCACAGGCAATTAATGGGGGAAGAACTTACGGGTTTGGGTATTAAAGAACTACAAAATCTGGAAAACCAACTGGAGATGAGTTTAAAGGGTGTCCGCATGAAAAAGGATCAAATTTTAACTAATGAGATTAAAGAACTACGCCAAAAGGGAAATATCATTCATCAAGAAAATGTTGAACTCTATCAAAAGATGGAGCAGATCCAAAAAGAAAATGCAGAGCTACAAAAGAAGGTTTATGAAGCAAGGAGTACAAATGAAGAAAATGCGGCATCCAATCCTTCTTACAACGTCAGAAATGGATATGATTCACTTGCATCTATCAGTCTCCAGCTAAGTCAGCCACAGTCTCAATACAAATACGGTGAACCATCAACCAAAGCAATGAAACTCGGATTGCAGCTGCATTAG

In the sequence, different colors refer to: black, the original sequence; blue: the selected *SP1* site.

**Protein sequence of *GmNMHC5* (WT, wild-type)**

MGRGKIAIRRIDNSTSRQVTFSKRRNGLLKKARELSILCDAEVGLMVFSSTGKLYDYASTSMKAVIERYNKLKEETHHLMNPASEEKFWQTEAASLRQQLQYLQECHRQLMGEELTGLGIKELQNLENQLEMSLKGVRMKKDQILTNEIKELRQKGNIIHQENVELYQKMEQIQKENAELQKKVYEARSTNEENAASNPSYNVRNGYDSLASISLQLSQPQSQYKYGEPSTKAMKLGLQLH

**CDS of GmNMHC5-*SP1***

ATGGGGAGAGGTAAGATTGCGATTCGAAGGATCGACAACTCCACTAGCCGGCAAGTGACTTTCTCAAAGAGAAGAAATGGATTGCTGAAGAAAGCTAGAGAATTATCAATTCTTTGTGATGCTGAAGTTGGATTGATGGTGTTCTCCAGCACTGGGAAGCTTTATGACTATGCAAGCACAAGCATGAAAGCGGTTATTGAACGCTACAACAAGCTAAAAGAGGAAACCCATCCTTCCAAGAGAAGGTAACTGTTTTTACACCATCACCCACTTGTACTGATTAAAAATGGGAACAAAAGGAGGAATACGAGTGGAGGTGACACTGTATGAGCCACGTTCCAAGATGTAAATGTTTCCGGCTTCAGAAGAGAAGTTTTGGCAGACAGAAGCAGCAAGCTTGAGGCAGCAGCTTCAGTACTTGCAAGAATGCCACAGGCAATTAATGGGGGAAGAACTTACGGGTTTGGGTATTAAAGAACTACAAAATCTGGAAAACCAACTGGAGATGAGTTTAAAGGGTGTCCGCATGAAAAAGGATCAAATTTTAACTAATGAGATTAAAGAACTACGCCAAAAGGGAAATATCATTCATCAAGAAAATGTTGAACTCTATCAAAAGATGGAGCAGATCCAAAAAGAAAATGCAGAGCTACAAAAGAAGGTTTATGAAGCAAGGAGTACAAATGAAGAAAATGCGGCATCCAATCCTTCTTACAACGTCAGAAATGGATATGATTCACTTGCATCTATCAGTCTCCAGCTAAGTCAGCCACAGTCTCAATACAAATACGGTGAACCATCAACCAAAGCAATGAAACTCGGATTGCAGCTGCATTAG

In the sequence, different colors refer to: black, the original sequence; blue: the selected *SP1* site; red, the mutation bases; green, the inserted bases.

**Protein sequence of *Gmnmhc5***

MGRGKIAIRRIDNSTSRQVTFSKRRNGLLKKARELSILCDAEVGLMVFSSTGKLYDYASTSMKAVIERYNKLKEETHPSKRR **Stop**
