# Supplementary material for: GmNMHC5, A Neoteric Positive Transcription Factor of Flowering and Maturity in Soybean
Source: Plants (Basel). 2020 Jun 25;9(6):792. doi: 10.3390/plants9060792 (PMC7356762; doi:10.3390/plants9060792)
Supplement: Supplementary file 1 [file plants-09-00792-s001.zip › Supplemental files/Table S6 Sequences of primers used in this study.docx]

Table S1 Sequences of primers used in this study

| **Primer Name** | **Primer Sequence** |
| --- | --- |
|  | **For Real-Time Quantitative PCR** |
| *qGmActin-F* | 5'-CGGTGGTTCTATCTTGGCATC-3' |
| *qGmActin-R* | 5'-GTCTTTCGCTTCAATAACCCTA-3' |
| *qGmNMHC5-F* | 5'-GGGTGTCCGCATGAAAAAGG-3' |
| *qGmNMHC5-R* | 5'-GGATTGGATGCCGCATTTTCT-3' |
| *qGmFT1a-F* | 5'-CCCAGCAAGGTAATCTGCAA-3' |
| *qGmFT1a-R* | 5'-ATCGATTATTTCCTCATACGTACA-3' |
| *qGmFT2a-F* | 5'-ATGCACCTAGCCCAAGTGAC-3' |
| *qGmFT2a-R* | 5'-TACACGGTCTCCCTACCCAG-3' |
| *qGmFT4-F* | 5'-GGCAACCACTGGAGAAGAGA-3' |
| *qGmFT4-R* | 5'-TGGAGCATGCACAATTTGTCT-3' |
| *qGmFT5a-F* | 5'-GCCTTACTCCAGCTGATACT-3' |
| *qGmFT5a-R* | 5'-GGCATGCTCTAGCATTGCAA-3' |
|  | **For overexpression-transgenic plants checking** |
| *GmNMHC5-F* | 5'-ATGGGGAGAGGTAAGATTGCGATT-3' |
| *GmNMHC5-R* | 5'-CTAATGCAGCTGCAATCCGAGTT-3' |
