# Supplementary figures and images for: GmNMHC5, A Neoteric Positive Transcription Factor of Flowering and Maturity in Soybean
Source: Plants (Basel). 2020 Jun 25;9(6):792. doi: 10.3390/plants9060792 (PMC7356762; doi:10.3390/plants9060792)

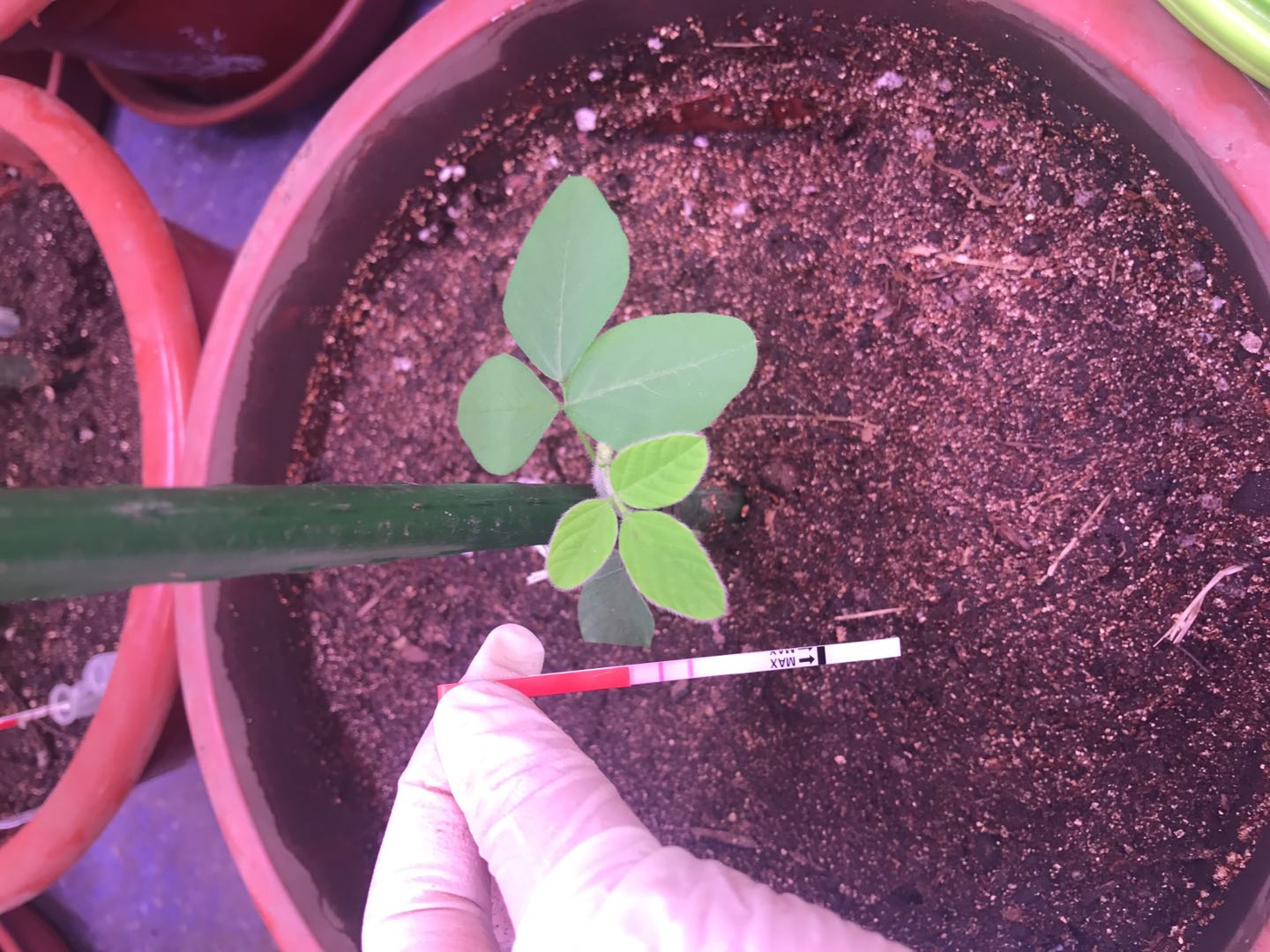

Supplement: Supplementary file 1 [file plants-09-00792-s001.zip › Supplemental files/Figure S2 Detection of the selectable marker gene bar by test strip.jpg]

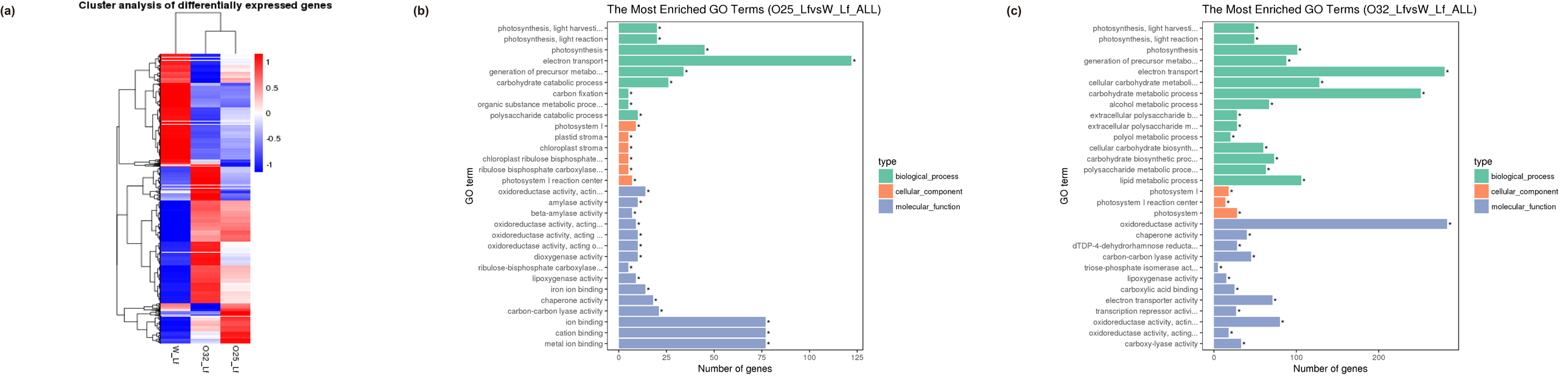

Supplement: Supplementary file 1 [file plants-09-00792-s001.zip › Supplemental files/Figure S3 The heat maps and GO analysis of the differentially expressed genes.tif]
